# Cancer-related cells and oncosomes in the liquid biopsy of pancreatic cancer patients undergoing surgery

**DOI:** 10.1038/s41698-024-00521-0

**Published:** 2024-02-15

**Authors:** Stephanie N. Shishido, Emmeline Lin, Nicholas Nissen, George Courcoubetis, Divya Suresh, Jeremy Mason, Arsen Osipov, Andrew E. Hendifar, Michael Lewis, Srinivas Gaddam, Stephen Pandol, Peter Kuhn, Simon K. Lo

**Affiliations:** 1 Convergent Science Institute for Cancer, Michelson Center, University of Southern California, Los Angeles, CA 90089 USA; 2 Pancreatic and Biliary Diseases Program, Cedars Sinai Medical Center, Los Angeles, CA 90048 USA; 3 Institute of Urology, Catherine & Joseph Aresty Department of Urology, Keck School of Medicine, University of Southern California, Los Angeles, CA 90033 USA; 4 Norris Comprehensive Cancer Center, Keck School of Medicine, University of Southern California, Los Angeles, CA 90033 USA; 5 IM Hematology Oncology, Cedars Sinai Medical Center, Los Angeles, CA 90048 USA; 6 Greater Los Angeles Veterans Affairs System, Los Angeles, CA 90073 USA; 7 Clark Atlanta University, Center for Cancer Research and Therapeutic Development, Atlanta, GA 30314 USA; 8 Department of Biomedical Engineering, Viterbi School of Engineering, University of Southern California, Los Angeles, CA 90089 USA; 9 Department of Aerospace and Mechanical Engineering, Viterbi School of Engineering, University of Southern California, Los Angeles, CA 90089 USA; 10 Department of Biological Sciences, Dornsife College of Letters, Arts, and Sciences, University of Southern California, Los Angeles, CA 90089 USA

**Keywords:** Pancreatic cancer, Pancreatic cancer

## Abstract

Pancreatic ductal adenocarcinoma (PDAC) has a five-year survival rate of less than 10% due to its late diagnosis, rapid metastasis, and chemotherapeutic resistance. For a small proportion (10–20%) of early-stage patients however, surgical resection of the pancreatic tumor offers the best chance for survival but the effect of surgery on disease dissemination is unknown. The primary objective of this study was to characterize cellular and acellular blood-based analytes in portal and peripheral blood before pancreatic manipulation, during tumor dissection and immediately after surgical resection to determine the effects of the surgery. This study used the non-enriching third generation High-Definition Single Cell Assay (HDSCA3.0) workflow to investigate heterogeneous circulating rare cell population in the blood. Blood from both sites taken before surgical manipulation of the pancreas had significantly greater incidence of total rare cellular and acellular analytes than normal donor samples. Post-surgery portal and peripheral blood had significantly greater incidence of specific cellular and acellular subtypes compared to the matched pre- and during-surgery samples. Our results reveal that in patients with PDAC liquid biopsy analytes are increased in both the portal and peripheral blood; portal blood contains a higher frequency of analytes than in the peripheral blood; total analytes in the portal and peripheral blood samples were significantly associated with the tumor volume and pathological T stage; and the surgical procedure increased the blood levels of circulating cellular and acellular analytes, but not Epi.CTCs or Mes.CTCs. This study demonstrates liquid biopsy’s utility in monitoring patients with PDAC with surgically resectable disease.

## Introduction

Pancreatic cancer, or pancreatic ductal adenocarcinoma (PDAC), has a five-year survival rate of less than 10%^1^ due to advanced-stage diagnosis^2^. For the small proportion (10–20%) of patients with early diagnosis prior to metastasis, resection of the pancreatic tumor offers the best chance for survival (five-year survival rate of 20%)^3^. However, recurrence is still common (71%) despite curative intent, and disease-free survival is rare, indicating that surgery alone may be inadequate treatment for PDAC^4^. The high incidence of metastatic diagnoses and poor outcome of many PDAC patients emphasizes the need for effective biomarkers to guide early diagnosis, real-time disease monitoring, and clinical decision-making in order to maximize patient survival. Currently, the only FDA-approved blood-based biomarker, Carbohydrate Antigen 19-9 has a high false positivity rate, low sensitivity, and low specificity, which is not useful for guiding patient care^5,6^. Therefore, finding more reliable biomarkers for PDAC is crucial for improving patient outcomes.

The liquid biopsy is a minimally-invasive approach to detecting circulating biomarkers in the blood. This technique is of increasing scientific and clinical interest because it is low-risk, simple to perform, and can be easily repeated to monitor the patient throughout their disease, making it a feasible implementation into cancer care. The liquid biopsy consists of a variety of analytes, including circulating tumor cells (CTCs) and other rare cells, platelets, extracellular vesicles, mRNA, protein, and cell-free DNA (cfDNA), which may be indicators of disease^7^. CTCs are cells released by the primary tumor and travel in the bloodstream, and have been of great interest as a biomarker due to their detectability. There is strong evidence that CTCs have prognostic significance in multiple cancer types such as breast^8,9^, bladder^10,11^, and colorectal^11–13^. Tumor-derived oncosomes and large extracellular vesicles have been detected in a variety of cancer types^14–18^, and have been shown to promote tumorigenesis^19–21^.

The current understanding of the clinical utility of the liquid biopsy in PDAC diagnosis and progression is limited. There are two main locations of blood collection for the liquid biopsy being explored in PDAC: the peripheral blood (PB) and portal vein blood (PoVB) which drains into the liver and is accessible during surgery or by using a guided endoscopic ultrasound (EUS)^22^. Studies using epithelial cell adhesion molecule (EpCAM)-enriched CTC-detection platforms such as CellSearch®^23–25^, CellMax (CMx®)^26^, and ClearCell®FX^27^ have reported that CTCs are detected at a significantly higher rate in PoVB than in PB in non-resectable PDAC. However, these methodologies have limited detection capability due to epithelial-marker enrichment approaches that overlook the mesenchymal phenotype found in some tumor cells^5,28^.

Few studies have used an enrichment-free detection method as an unbiased approach to analyze all cells in the sample. Marrinucci et al. utilized a “no cell left behind” approach and detected at least 5 CTCs per mL in 50% of a cohort of eighteen metastatic PDAC patients, with two presenting over 50 CTCs per mL blood^29^. However, out of the three cancer types studied (breast, prostate, and pancreatic), PDAC had the lowest frequency of detection, highlighting the challenges of using CTCs as a biomarker for PDAC. A further challenge is detecting analytes in early-stage disease, in which clinical intervention would be most critical for patient survival.

This study presents the utility of a comprehensive, enrichment-free liquid biopsy approach to detect circulating biomarkers in patients with early-stage, resectable PDAC. Previously, we have used the third-generation High-Definition Single Cell Assay (HDSCA3.0) to detect different types of rare cells, such as CTCs or circulating endothelial cells, and acellular events such as oncosomes, in cancer patients^16–18,30^. The primary objective of the present study was to characterize these blood-based biomarkers in the context of the time before, during and after surgical resection in both PoVB and PB to determine if surgical resection affects the cancer related biomarkers. Circulating biomarkers were correlated with clinical data metrics such as treatment status, tumor staging, and post-surgical progression. Ultimately, this study aims to understand the efficacy of surgical resection through the use of a liquid biopsy, which shows promise in PDAC patient care to guide clinical decision-making and improve patient outcomes.

## Results

### Patient and sample information

A total of 20 patients with early-stage PDAC were included in this study, each providing up to 10 blood samples from the different locations and times. Patient #6 received no PB draws, and Patient #9 received no PoVB draws. Clinical and demographic data were collected and is provided in Table 1. In this cohort, 4 patients did not receive any chemotherapy or radiation treatment, 3 patients received neoadjuvant therapy (Folfironox and/or radiotherapy), 11 patients received adjuvant therapy (Folfironox, Stereotactic Body Radiation Therapy, and/or Gemcitabine), and 2 patients were missing data. A complete blood cell count was taken for each PDAC patient for each blood sample. The average WBC count among PDAC patients was 7.49 (range = 2.7–17.7; median = 6.7) million cells/mL blood. Among NDs, the average WBC count was 6.3 (range = 3.0–11.6; median = 6.1) million cells/mL.

**Table 1:** Clinical and demographic data of PDAC patients

Variable	Category	Value *n* (%)
Age mean (range)		66.45 (61–82)
Sex	Male	9 (45%)
	Female	11 (55%)
Race	White/Caucasian	14 (70%)
	Black/African American	3 (15%)
	Other	3 (15%)
Smoker	Yes	8 (40%)
	No	12 (60%)
Alcohol Consumption	Yes	5 (25%)
	No	15 (75%)
Familial History of Cancer	Yes	13 (65%)
	No	7 (35%)
Tumor Grade	Poorly Differentiated	5 (25%)
	Moderately Differentiated	13 (75%)
	NA	2 (10%)
Tumor Volume mean (range) (mm^2)	*n* = 8	48.61 (4.86–184.9)
	NA (*n* = 12)	
Pathological T stage	pT0	1 (5%)
	pT1	8 (40%)
	pT2	8 (40%)
	pT3	1 (5%)
	pT4	1 (5%)
	NA	1 (5%)
Pathological N stage	pNX	5 (25%)
	pN0	7 (35%)
	pN1	7 (35%)
	NA	1 (10%)
Chemotherapy	None	5 (25%)
	Neoadjuvant	3 (15%)
	Adjuvant	11 (65%)
	NA	1 (10%)

The pathologic tumor (T) stages are as follows: pT0 – No evidence of primary tumor, pT1 – the diameter of the cancer is 2 centimeters (or less) and has not grown outside the pancreas, pT2 – the diameter of the cancer is larger than 2 centimeters and has not gown outside the pancreas, pT3 – the cancer has spread outside the pancreas into nearby surrounding structures and is not found in major blood vessels or nerves, pT4 – the cancer has grown beyond the pancreas and into nearby large blood vessels or nerves. The pathological lymph nodes metastases (N) stages are as follows: pNX – regional lymph nodes cannot be assessed, pN0 – the cancer has not spread to regional lymph nodes, pN1 – the cancer has spread to regional lymph nodes.

*NA* Not available.

### Liquid biopsy analysis of PDAC patients and NDs

**Fig. 1:**
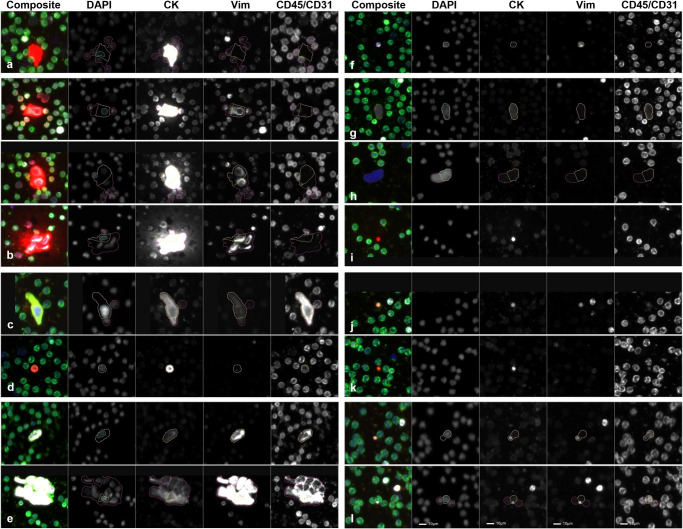
Representative gallery of rare events detected in samples collected from patients diagnosed with PDAC. For each rare event the composite image is provided followed by the individual biomarker fluorescent channels which are indicated by the headers. **a** Epi.CTC; **b** Mes.CTC (top: single cell; middle: cluster of 2; bottom: cluster of 4); **c** DAPI|CK|Vim|CD45/CD31; **d** DAPI|CK|CD45/CD31; **e** DAPI|Vim|CD45/CD31 (top: single cell; bottom: cluster); **f** DAPI|Vim; **g** DAPI|CK|CD45/CD31; **h** DAPI-only; **i** CK oncosome; **j** CK|Vim oncosome; **k** CK|CD45/CD31 oncosomes; **l** CK|Vim|CD45/CD31 oncosome (top: distal, bottom: proximal). Blue: DAPI, Red: CK, White: Vim, Green: CD45/CD31. Images taken at 100X magnification. Scale bar = 10 µm.

Among PDAC patients, we identified 8 cellular categories with nuclear DAPI expression and variable expression of the other three protein biomarkers, which are shown as a representative gallery in Fig. 1a–h. Oncosomes were classified by their lack of DAPI signal and a positive CK signal, with variable expression in the other two biomarker channels, as well as round morphology (Fig. 1i–l).

**Fig. 2:**
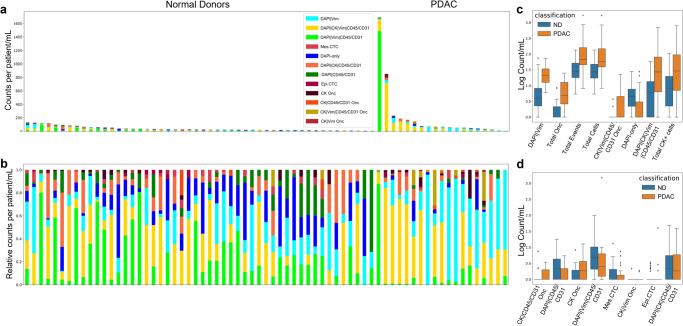
Rare-event detection in samples collected before surgery from PB of PDAC patients using HDSCA3.0, compared to NDs. **a** Enumeration and (**b**) frequency of each rare event by channel-type classification. Logarithmic box and whisker plots of the channel-type rare events/mL that are (**c**) statistically significant and (**d**) non-significant between PDAC and ND samples. Center line represents median, box limits represent upper and lower quartiles, whiskers represent 1.5x interquartile range, and points represent outliers.

To determine if there are cancer related liquid biopsy analytes present in the circulation of PDAC patients, PB samples taken prior to surgical resection were compared to ND controls. PDAC draws had significantly greater incidence of total rare events (cellular and acellular combined), total rare cells, and total CK-expressing cells than ND (*p*-value = 0.0005, 0.0014, and 0.025, respectively’; Fig. 2). PDAC draws also had significantly greater DAPI|Vim cells and DAPI|CK|Vim|CD45/CD31 cells than ND (*p*-value = 0.00000013 and 0.0086respectively). This indicates that specific rare cell populations detected in the PB of PDAC patients are unique biomarkers of disease. The DAPI-only cells were the only rare cell category that was obsereved at a higher prevalence in the ND than PDAC (*p*-value = 0.0069). PDAC and ND analyte incidence is provided in Supplementary Table 1. Comparison between PDAC and ND samples is provided in Supplementary Table 2.

We further explored the significance of the oncosome population detected in PDAC compared to the ND cohort. PDAC samples across all timepoints and locations had a median of 7.29 total oncosomes/mL (range = 0–1828.28; mean = 64.13). Oncosomes were detected either distally (stand-alone: median = 4.67; range = 0.49–405.09; mean = 31.31 oncosomes/mL; 64.76%) or in close proximity to a cell (median = 3.24; range = 0.35–278.58; mean = 22.98 oncosomes/mL; 35.24%). The potential implications of an oncosome being associated with a cell (in proximity) is currently unknown. Comparison of pre-surgical PB PDAC to ND draws revealed significant differences in oncosome incidence. PB PDAC draws had significantly greater total oncosomes and CK|Vim|CD45/CD31 oncosomes than ND samples (*p*-value = 0.00002 and 0.004, respectively) (Fig. 2). Oncosomes were detected at a higher incidence in the PDAC patient samples compared to the ND samples suggesting this analyte may be a critical biomarker for disease monitoring.

### Rare cell characterization

**Fig. 3:**
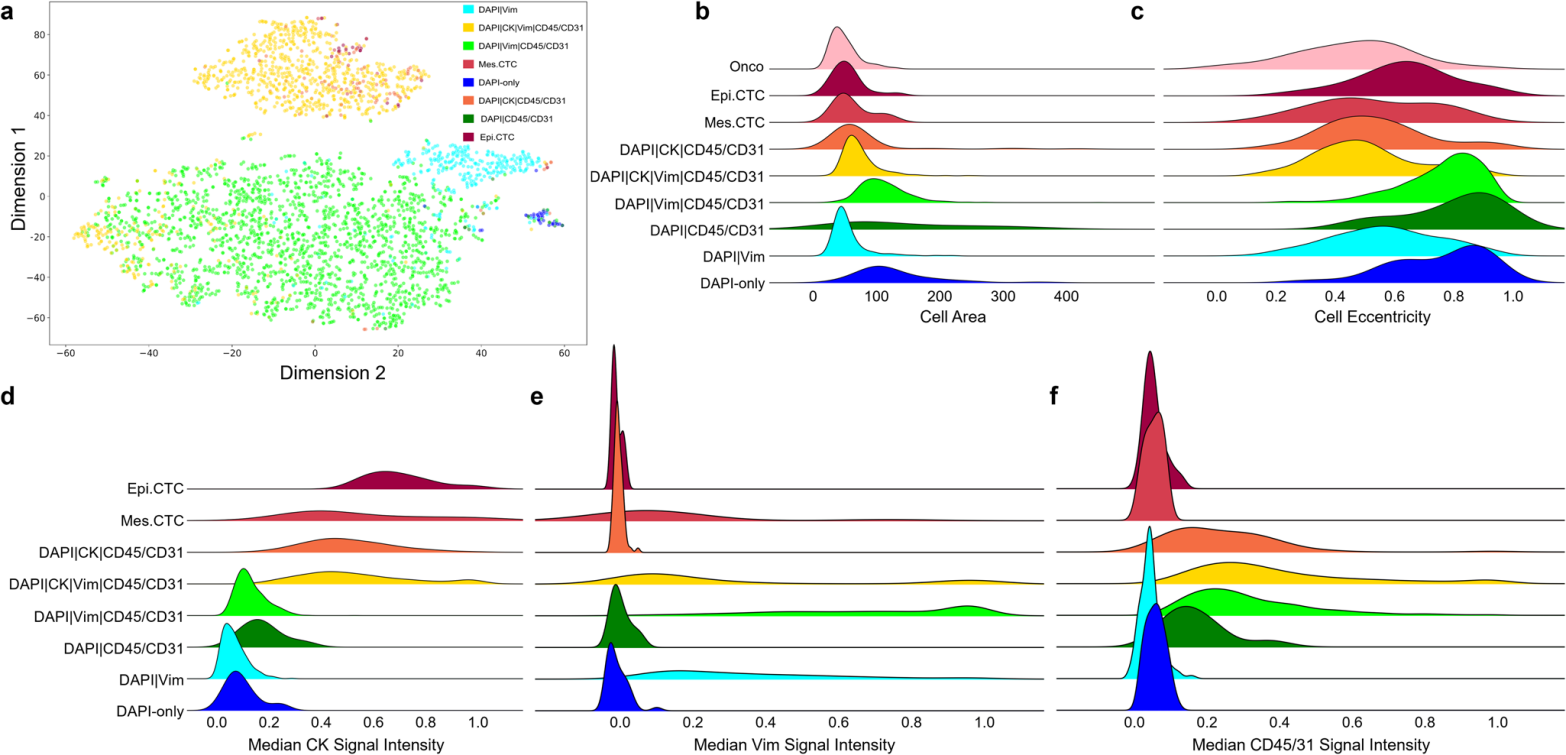
Morphometric analysis of individual cells detected by HDSCA3.0 in all PDAC samples. **a** tSNE plot of morphologically heterogeneous rare cells. Each point represents a single event and is color-coded according to its channel-type classification. Also visualized are the probability density distributions for select morphometric parameters across channel-type classifications of (**b**) event area, (**c**) event eccentricity, (**d**) median CK signal intensity, (**e**) median Vim signal intensity, and (**f**) median CD45/CD31 signal intensity.

To investigate the heterogeneity of the rare cell classifications detected in PDAC blood samples the cellular morphometrics were analyzed using a dimensionality reduction algorithm and a clustering algorithm. The area, eccentricity, and the median intensity of the four IF channels of the individual cells were compared in a two-dimensional cellular tSNE (Fig. 3a) in which each rare cell is represented with a single point and color-coded according to channel-type classification. This shows that specific phenotypic classifications cluster separately from the other rare cells (i.e., DAPI|Vim, DAPI) suggesting a more homogenous population of cells, while other classifications form mixed clusters (i.e., DAPI|CK|Vim|CD45/CD31 and DAPI|Vim|CD45/CD31, DAPI|CK|Vim|CD45/CD31 and Epi.CTC) suggesting these are morphologically related. The distribution of cellular area, eccentricity, and median channel intensities for each channel-type classification is further plotted in Fig. 3b–f. From these density plots, we can observe the morphological heterogeneity of the channel-type classifications. As an example, the Mes.CTC population was found to have two distinct size groups (<100 and >100) while also varying in cellular shape (eccentricity) and CK expression. The Mes.CTCs were more variable in size, shape, and signal intensity than the Epi.CTCs. Taken together, Fig. 3 indicates that the channel-type classified cellular populations detected in the PDAC cohort are composed of multiple cellular populations that vary in size, shape, and signal intensity. Further analysis may elucidate the different biological cell types present in each classification and provide a better understand of the predictive potential of individual analytes or a series of analytes.

### Rare event characterization according to time-of-draw

**Fig. 4:**
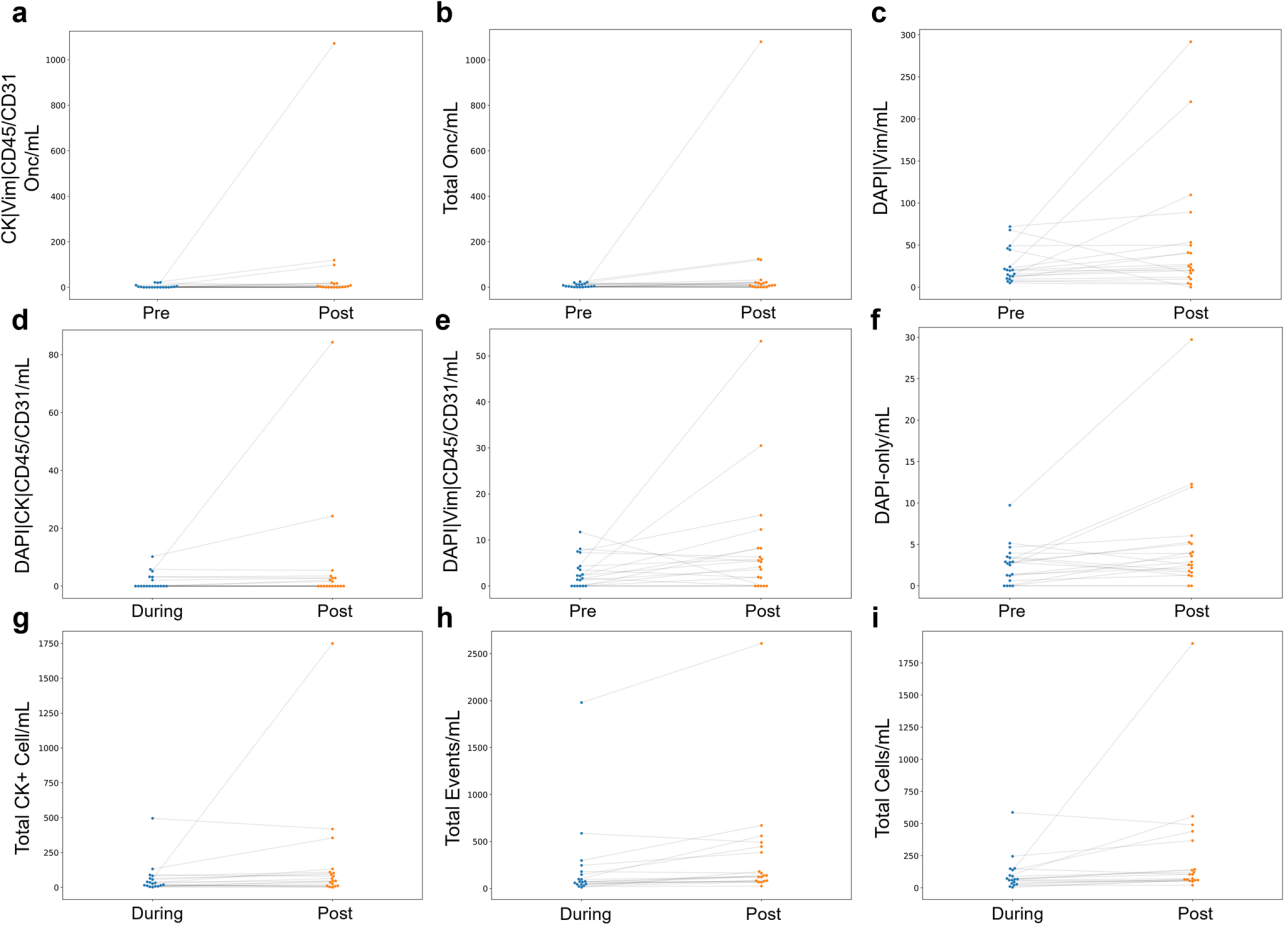
Graphical representation of the difference in channel-type rare events/mL between time points. Matched analysis of significant rare events for (**a**–**d**) PB and (**e**–**i**) PoVB.

To determine if surgery affects the circulating analytes in PDAC patients, we compared the liquid biopsy analytes before, during, and after surgical removal of the tumor (Fig. 4 and Table 2). There were no significant differences in rare event enumeration or frequency between Pre- and During-surgery timepoints. Specific channel-type classifications of rare analytes found in PB were observed at a higher incidence in the Post-surgery sample (Fig. 4a). Post-surgery PB draws had greater incidence of DAPI|Vim (*p*-value = 0.023), total oncosomes (*p*-value = 0.022) and CK|Vim|CD45/CD31 oncosomes (*p*-value = 0.020) than Pre-surgical PB draws. Compared to the During-surgical draw, the Post-surgical draw had a greater number of DAPI|CK|CD45/CD31 cells (*p*-value = 0.050). This does suggest that immediately after surgical resection there are more tumor associated analytes circulating in the PB. All other PB analytes were not significantly different between timepoints (Fig. 4b).

**Table 2:** Liquid biopsy analytes with statistically significant differences detected between timepoints

Draw site	Timepoints	Quantity of interest	*p*-value
PB	Pre vs Post	CK|Vim|CD45/CD31 Onc	0.0199
PB	Pre vs Post	Total Onc	0.0217
PB	Pre vs Post	DAPI|Vim	0.0230
PB	During vs Post	DAPI|CK|CD45/CD31	0.0499
Portal	Pre vs Post	DAPI|Vim|CD45/CD31	0.0437
Portal	Pre vs Post	DAPI-only	0.0442
Portal	During vs Post	Total Events	0.0016
Portal	During vs Post	Total Cells	0.0077
Portal	During vs Post	Total CK+ Cells	0.0268

The analysis was conducted on the counts per mL of blood analyzed for matched samples. The Wilcoxon signed-rank test was used, and the corresponding *p*-value is reported for PB and for PoVB across all time point combinations. There were no significant differences in liquid biopsy analytes between Pre- and During-surgery collection timepoints.

Differences in frequencies of channel-type classifications found in PoVB of PDAC patients were also observed between different times of sample collection (Fig. 4c). Compared to Pre-surgery PoVB draws, Post-surgical PoVB draws had a higher incidence of frequency of DAPI-only (*p*-value = 0.044) and DAPI|Vim|CD45/CD31 (*p*-value = 0.044) cells. Post-surgical PoVB draws had a greater total rare event (*p*-value = 0.002), total rare cell (*p*-value = 0.008), and total CK-expressing cell (*p*-value = 0.027) counts than Dur-surgery samples. There was no significant difference in incidence of the other PoVB analytes between timepoints of collection (Fig. 4d). Interestingly, there is no concordance between analytes determined to differ by time of collection in the PoVB and PB. This suggests that the two anatomical locations differ in the presence of circulating biomarkers by time. Overall, this data suggests that immediately after surgical resection there are more tumor associated analytes circulating in the PB and PoVB, but not specifically Epi.CTCs or Mes.CTCs.

Pre-, During-, and Post-surgery analyte incidence is provided in Supplementary Table 1. Comparison between timepoints matched by sampling site is provided in Supplementary Table 2.

### Rare event characterization according to anatomical location

To determine if the PoVB was more sensitive in detecting cancer-related biomarkers and thus predictive value for clinical outcomes, we compared the liquid biopsy analytes by anatomical location (PB vs. PoVB), matched by time point. For draws taken Pre- or During-surgery, there was no difference in the liquid biopsy analytes detected in the PoVB and PB (Supplementary Table 2). This suggests concordance in the liquid biopsy profile of the PB and PoVB at these timepoints.

**Fig. 5:**
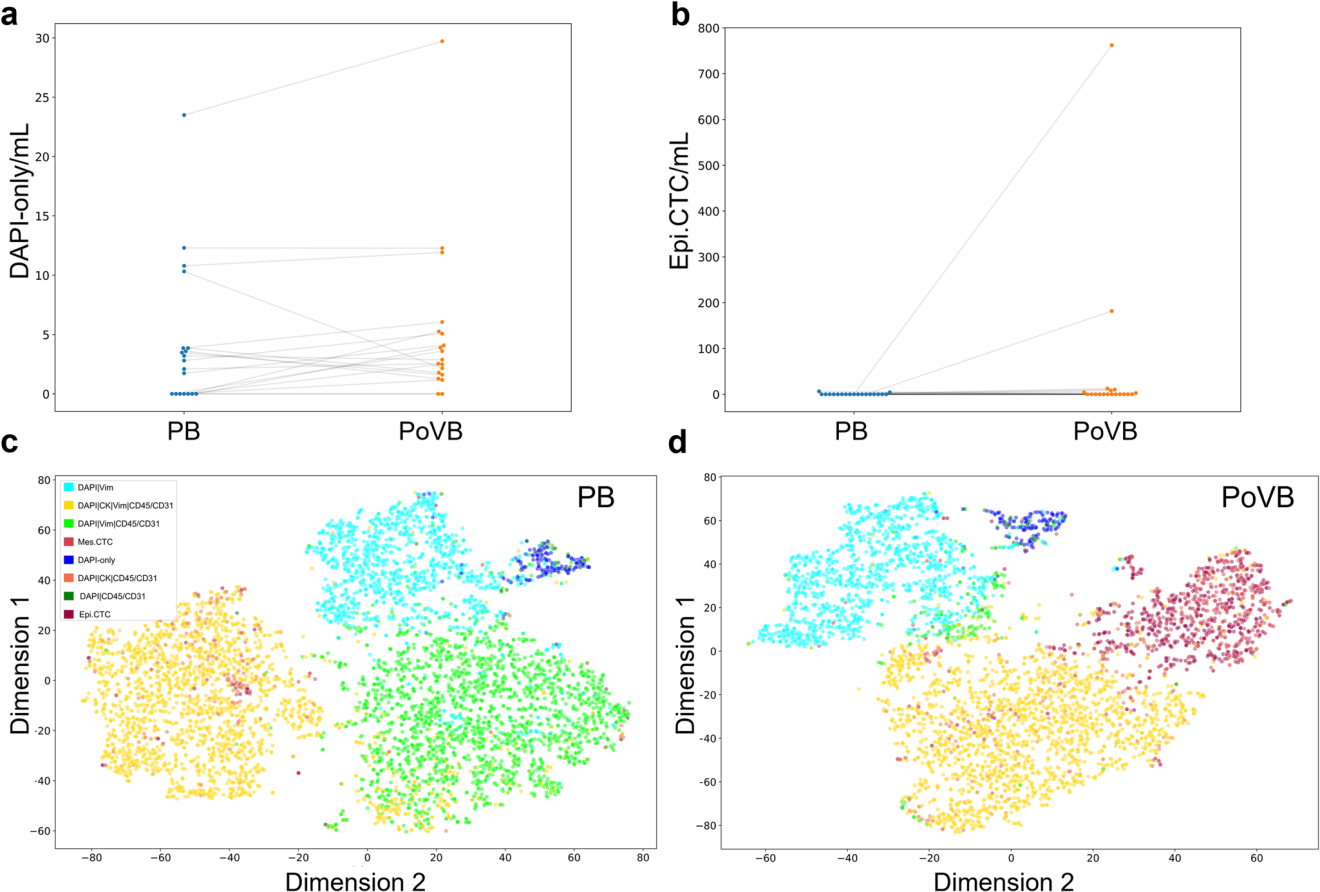
Graphical representation of the different channel-type rare events/mL between anatomical locations matched by time point. **a**, **b** Matched analysis of significant rare events for PB vs. PoVB for. Morphology tSNE plot of rare cells in (**c**) PB and (**d**) PoVB at all timepoints. D: DAPI, V: Vim, CD: CD45/CD31.

Analysis at the Post-surgical time point revealed that PoVB draws had a greater Epi.CTC count than PB draws (*p*-value = 0.03), as well as a greater DAPI-only cell count (*p*-value = 0.04) (Fig. 5a, b). Interestingly, despite the presence of Epi.CTCs in the PoVB draw, these cells are not present in the peripheral circulation. The tSNE plots of the individual rare cells detected from each location across timepoints shows the Epi.CTC population is more prevalent in PoVB draws and forms a distinct cluster from the other rare cells indicating a unique morphology and phenotype (Fig. 5c, d). The significance of the DAPI-only cell phenotype is currently unknown.

PB and PoVB analyte incidence is provided in Supplementary Table 1. Comparison between anatomical locations matched by timepoint is provided in Supplementary Table 2.

### Correlation with clinical data

To further explore the potential clinical significance of the liquid biopsy analytes detected in PDAC patient samples, correlations between PDAC Pre-surgery time point for both PB and PoVB rare event occurrence and clinical data elements were analyzed using the Wilcoxon rank sum test for categorical variables and Spearman’s rank correlation for continuous and ordinal variables. All significant correlations are presented in Table 3. Tumor volume and pathological T stage were the most common variables to correlate with the liquid biopsy analytes. Analysis of Pre- and Post-surgery PB and PoVB analytes for patients that received neoadjuvant therapy versus those that did not, revealed a lack of statistically significant differences.

**Table 3:** Significant correlations between the clinical/demographic variables and the rare events identified by HDSCA3.0 in the liquid biopsy samples collected Pre-surgery from PDAC patients

Sample	Type of variable	Clinical/Demographic variable	HDSCA analysis output	*p*-value	Correlation statistic
PB	Ordinal	pT stage	DAPI|CK|CD45/CD31	0.0099	0.59
PoVB	Continuous	Tumor Volume	Total Cells	0.0102	0.83
PB	Continuous	Tumor Volume	Total CK+ Cells	0.0137	0.86
PoVB	Continuous	Tumor Volume	DAPI|Vim	0.0149	0.81
PB	Ordinal	pT stage	Total CK+ Cells	0.0154	0.56
PoVB	Categorical	Gender	Total Events	0.0167	−2.39
PoVB	Ordinal	pT stage	Total CK+ Cells	0.0189	0.53
PoVB	Continuous	Tumor Volume	DAPI|CK|Vim|CD45/CD31	0.0208	0.79
PoVB	Categorical	Prior Therapy (Yes/No)	DAPI-only	0.0257	−2.23
PB	Categorical	Gender	DAPI|Vim|CD45/CD31	0.0258	−2.23
PoVB	Ordinal	pT stage	DAPI|CK|Vim|CD45/CD31	0.0309	0.50
PB	Continuous	Tumor Volume	Total Events	0.0362	0.79
PB	Continuous	Tumor Volume	Total Cells	0.0362	0.79
PB	Ordinal	pT stage	DAPI|CK|Vim|CD45/CD31	0.0381	0.49
PoVB	Categorical	Smoking	Total Onc	0.0409	−2.04
PoVB	Ordinal	pN stage	D|CK	0.0424	−2.03
PoVB	Ordinal	pT stage	DAPI|CK|CD45/CD31	0.0446	0.47
PoVB	Continuous	Tumor Volume	Total Events	0.0465	0.71
PB	Categorical	Gender	Total Events	0.0475	−1.98
PoVB	Categorical	Race	DAPI|CK|Vim	0.0478	1.98
PoVB	Categorical	Race	DAPI|CK	0.0478	1.98

Among PoVB draws, there was a significant positive correlation between tumor volume and incidence of total rare events (*p*-value = 0.047), total rare cells (*p*-value = 0.01), DAPI|Vim (*p*-value = 0.01), and DAPI|CK|Vim|CD45/CD31 (*p*-value = 0.02) cells. There was also a significant positive correlation between pathological T stage and total CK-expressing cells (*p*-value = 0.019), DAPI|CK|Vim|CD45/CD31 (*p*-value = 0.03), and DAPI|CK|CD45/CD31 (*p*-value = 0.04) cells within PoVB draws. In the PB, significant positive correlations were also identified between liquid biopsy analytes and tumor volume or pathological T stage. Specifically, tumor volume correlated with the incidence of total rare events (*p*-value = 0.036), total rare cells (*p*-value = 0.036), and total CK+ events (*p*-value = 0.014). Pathological T stage correlated with the number of total CK+ cells (*p*-value = 0.015), DAPI|CK|Vim|CD45/CD31 (*p*-value = 0.038), and DAPI|CK|CD45/CD31 (*p*-value = 0.0099) cells.

As more analytes were found to be correlated in the PoVB sample, this supports that the closer anatomical location of collection provides more information about the tumor. Interestingly, the total rare events and total rare cell count in the PoVB and PB were significantly associated with the tumor volume and pathological T stage, indicating that the overall count of circulating analytes is indeed related to the presence of a malignant tumor in the patient’s body. All correlation analyses of liquid biopsy analytes in PDAC compared to pathological T stage and tumor volume are provided in Supplementary Table 3.

At the time of this manuscript preparation most (*n* = 13) of our patients were alive.

## Discussion

In this cohort we have detected twelve heterogeneous liquid biopsy analytes (8 cellular, 4 oncosome) in the blood of PDAC patients receiving surgical resection, demonstrating the utility of a non-enriching liquid biopsy in both early detection and prognostic workup of PDAC. The key findings from this cohort are as follows: 1) liquid biopsy analytes are detected at a higher incidence in the PB of patients diagnosed with localized PDAC than NDs; 2) the time point of sample collection in relation to surgical manipulaton and tumor resection significantly affects the detectable analytes; 3) the anatomical location of blood draw is significantly associated with different rare event profiles; and 4) pancreatic tumor volume and pathological T stage are significantly associated with cellular biomarkers in both draw locations.

The non-enriching HDSCA3.0 approach allows for the identification of novel acellular biomarkers such as the four different CK-expressing oncosome classifications. Total rare cells and oncosomes were detected in PDAC PB samples at a significantly higher count than in ND samples (median 57.0 vs. 11.5 cells/mL and 3.90 vs. 0 oncosomes/mL, respectively). The majority of the rare cells are hypothesized to be cancer related. The oncosomes may be used to supplement CTCs and circulating tumor DNA (ctDNA) as biomarkers for characterization of the heterogeneity of PDAC tumors. Both rare cells and oncosomes are integral for distinguishing PDAC patients from NDs and may be useful for diagnostic workup of PDAC.

This study also demonstrates that detection of rare events, specifically Epi.CTCs, is greater in the PoVB than PB draws at the Post-surgical time point. While this difference has been confirmed in metastatic PDAC patients^27^ research on the incidence of CTCs in early-stage disease is limited. Song et al. reported no significant difference in the number of CTCs collected intraoperatively from the two locations, while Poruk et al. reported that peripheral venous blood had significantly greater Epi.CTC count than PoVB when collected prior to surgical incision^31,32^. Multiple other studies^26,33,34^ focused on intraoperative collection all reported greater CTC counts in PoVB than in PB of PDAC patients. This difference in CTC presence by anatomical location may be explained by the hepatic filtration effect: blood from the pancreas flows to the liver via the portal vein, which may remove CTCs or other tumorigenic events from the blood, before entering into peripheral circulation^24^. Since PoVB is collected closer to the site of the primary tumor and does not undergo filtration, PoVB CTCs are much less rare than peripheral CTCs^35^. Furthermore, CTC detection in PoVB has been found to be associated with the occurrence of latent metastases^36^ and worse overall and progression-free survival^33^ among PDAC patients. This indicates that the liquid biopsy taken from PoVB may have prognostic utility and can be used to inform decisions regarding adjuvant chemotherapy and postoperative oncological surveillance^36^. In the study presented here, we confirm that PoVB contains more analytes than PB.

Draws taken immediately after tumor resection had a greater frequency of specific analytes than draws taken before pancreatic manipulation and during surgery in both PB and PoVB, but differences in Epi.CTC and Mes.CTC counts specifically were not significant. Our data demonstrates that the liquid biopsy can detect additional analytes beyond the conventional CTC that may be clinically useful in predicting surgery outcome, but further investigation of their exact mechanisms is warranted. Previous studies have hypothesized that surgical manipulation of the tumor may cause CTCs to be released into the bloodstream, leading to elevated CTC counts and contributing to future recurrence^37^. Similarly, White et al. found no significant increase in CTC count between Pre- and Post-surgical timepoints in both PB and PoVB^38^ of patients with neoadjuvant therapy. The results presented here indicate that surgery does not significantly affect the CTC populations in circulation, despite there being an increase in rare analytes (i.e., oncosomes and other rare cells) Post-surgical resection. This suggests that surgical resection does not cause dissemination of disease (i.e., CTCs with metastatic potential), however clinical follow up to monitor patient outcomes will further determine the efficacy of surgery and the association with the liquid biopsy analytes measured here.

Tumor volume and pathological T stage were found to be significantly associated with incidence of total cellular events and total CK-expressing cells, respectively, in both PB and PoVB draws collected before surgery. Previous research has reported a correlation between CTC frequency and tumor size. Nagrath et al. found that over the course of a gemcitabine-based treatment for nine metastatic PDAC patients, tumor volume decreased alongside CTC counts^39^. Similarly, in a study on three stage IV metastatic PDAC patients, Sheng et al. found that CTC number decreased with continuation of treatment and correlated proportionally with tumor size^40^. While we found no correlation between tumor volume and specifically Epi.CTC or Mes.CTC frequency, we demonstrate that a heterogeneous cohort of biomarkers, which include both CTCs and other cellular and acellular events, can reflect tumor volume for non-metastatic, early-stage PDAC patients. This indicates the utility of the liquid biopsy as a powerful clinical tool for monitoring patient response to cancer treatment and cancer progression/tumor growth.

A limitation of this present study is the small sample size due to the nature of the study design. The twenty patients analyzed here displayed heterogeneity in quantity and identity of rare cell types, as well as in clinical care received. A larger sample size is needed to better identify, understand, and establish correlations between cellular events, surgical parameters, and clinical history in PDAC patients. Further, we recognize that each channel-type classification likely contains multiple biological cell types, as shown by the heterogenous morphometric analysis, which needs to be investigated further. The current study suggests utility of specific cellular classification, which further stratification of cellular types by morphology may provide additional predictive utility or may be associated with additional clinical characteristics. Additionally, molecular characterization of the genome and proteome of both circulating cellular and acellular analytes can help to further explain the association between circulating biomarkers and PDAC tumorigenesis and metastasis. While the role of Epi.CTCs and Mes.CTCs has been well-studied, understanding of the roles of the other cellular subtypes (in either facilitating tumor microenvironment or transporting tumor material) is limited. The HDSCA3.0 workflow allows for downstream analysis via targeted multiplexed proteomics and single cell genomic analysis to aid in further biomarker characterization.

In conclusion, we demonstrate that the non-enriching liquid biopsy approach can be successfully used to identify circulating tumor biomarkers in the bloodstream of PDAC patients undergoing surgical resection of the primary tumor. Using the liquid biopsy we demonstrate the clinical significance of a heterogeneous group of biomarkers, which include both CTCs and other cellular and acellular events, which can reflect tumor volume and may be useful for diagnostic workup of non-metastatic, early-stage PDAC or postoperative oncological surveillance. While further research is necessary to understand the predictive power of these analytes in respect to tumor development and cancer prognosis, the liquid biopsy is a promising clinical tool for early stage PDAC patients.

## Methods

### Study design

Blood samples were collected from 20 localized PDAC patients undergoing surgical resection of their pancreatic tumor. The study was conducted according to the guidelines of the Declaration of Helsinki and approved by the Institutional Review Board (or Ethics Committee) of the University of Southern California Keck School of Medicine (protocol HS-20-00974 approved on 2 February 2021) and Cedars-Sinai Medical Center (IRB #4201). Patient recruitment was done in accordance with protocols from the Institutional Review Board, and all patients provided written informed consent prior to enrollment into the Biomarker Studies for Pancreatic Diseasesat Cedars-Sinai Medical Center.

Blood samples were collected between January 2021 and January 2022 on the day of surgical resection of pancreatic tumor. The two anatomical locations of sample collection were PB and PoVB. Portal vein blood samples were collected with direct needle puncture of the main portal vein. Collection time points included before surgical manipulation of the pancreas (Pre-), during surgery and immediately after the pancreatic tissue had been transected (During-), and after the tissue containing the cancer had been removed but before abdominal wall closure (Post-) for both PB and PoVB. Information about medical history, tumor staging, and treatment status were collected for each patient. Additionally, 50 normal donor (ND) PB samples from individuals with no known pathology were procured from Epic Sciences (San Diego, California, USA) and analyzed for a comparative analysis.

### Blood sample processing

All blood samples were collected in 10 mL blood collection tubes (Cell-free DNA, Streck, La Vista, NE, USA) and processed by the Convergent Science Institute in Cancer (CSI-Cancer) at the University of Southern California within 48 h. Samples first underwent red blood cell lysis in isotopic ammonium chloride solution. The remaining nucleated cell fraction was then plated on custom glass slides at approximately 3 million cells per slide (Marienfeld, Lauda, Baden-Württemberg, Germany) and blocked with 7% BSA before being placed in long-term cryostorage at −80 °C.

### Blood sample staining and imaging

Each patient test sample utilized two slides, with an average of 0.94 mL blood analyzed per test. The slides were stained at room temperature using the IntelliPATH FLXTM autostainer (Biocare Medical LLC, Irvine, CA, USA) according to the Landscape immunofluorescence (IF) protocol as described previouslye^16–18,30^. Briefly, after fixation with 2% paraformaldehyde, slides are incubated with 2.5 ug/mL of a mouse IgG1 anti-human CD31:Alexa Fluor® 647 mAb (clone: WM59, MCA1738A647, BioRad, Hercules, CA, USA) and 100 ug/mL of a goat anti-mouse IgG monoclonal Fab fragments (115-007- 003, Jackson ImmunoResearch, West Grove, PA, USA). Then, the slides were permeabilized using 100% cold methanol, followed by an antibody mixture consisting of mouse IgG1/Ig2a anti-human cytokeratins (CKs) 1, 4, 5, 6, 8, 10, 13, 18, and 19 (clones: C-11, PCK-26, CY-90, KS-1A3, M20, A53-B/A2, C2562, Sigma, St. Louis, MO, USA), mouse IgG1 anti-human CK 19 (clone: RCK108, GA61561-2, Dako, Carpinteria, CA, USA), mouse anti-human CD45:Alexa Fluor® 647 (clone: F10-89-4, MCA87A647, AbD Serotec, Raleigh, NC, USA), and rabbit IgG anti-human vimentin (Vim or V) (clone: D21H3, 9854BC, Cell Signaling, Danvers, MA, USA). Lastly, slides were incubated with Alexa Fluor® 555 goat anti-mouse IgG1 antibody (A21127, Invitrogen, Carlsbad, CA, USA) and 4′,6-diamidino-2-phenylindole (DAPI; D1306, ThermoFisher) prior to coverslipping with a glycerol-based aqueous mounting media. Automated high-throughput fluorescence scanning microscopy was conducted at 100× magnification, collecting 2304 frames per slide^16–18,29,30^

### Rare event detection and classification

OCULAR (Outlier Clustering Unsupervised Learning Automated Report), a custom computational algorithm, was used to automatically analyze the microscopy images to identify and detect rare cell candidates from every slide based on 761 quantitative morphometric parameters based derived from nuclear and cytoplasmic morphometry and biomarker expression (CK, Vim, and CD45/CD31) from the four-channel IF assay (DAPI, AlexaFluor® 488, AlexaFluor® 555, AlexaFluor® 647)^16–18,30^. In addition to OCULAR, the identified events were screened by trained analysts using a manual reporting process for data reduction to confirm signal intensity and distribution, as well as distinct morphology. There were twelve categories of rare events (8 cellular, 4 oncosome) based on different combinations of the four IF marker expressions. The nomenclature for each event channel-type classification utilizes the positive expression of each biomarker, with CD45 and CD31 in a single flourescent channel and refered to as “CD45/CD31”. The total rare event counts specify the combination of cellular and accellular (ocosomes) events detected.

Epithelial-like CTCs (Epi.CTCs) were classified as cells with a distinct nucleus, CK-positive, Vim-negative, and CD45/CD31-negative^17,18,30^. Epi.CTCs with Vim expression were classified as mesenchymal-like CTCs (Mes.CTCs). Additional rare event categories of interest included two candidate CTCs: 1) triple (CK|Vim|CD45/CD31) positive CTCs with expression in all four channels, and 2) CK|CD45/CD31 positive CTCs. The other three rare cell classifications were: Vim|CD45/CD31, Vim-positive only, and CD45/CD31-positive only. White blood cell (WBC) counts of whole blood in each slide were determined automatically (Medonic M-series Hematology Analyzer, Clinical Diagnostic Solutions Inc., Fort Lauderdale, FL, USA) to calculate the actual amount of blood analyzed per test, allowing results to be displayed as fractional values of events/mL. In this study acellular round CK-positive events that lack a nuclear structure (DAPI-negative) with variable Vim and CD45/CD31 expression were defined as as oncosomes, a type of large extracellular vesicle^15–18^. Oncosomes were detected individually or adjacent to nucleated common cells and manually classified and confirmed. Manual data reduction was utilized to remove any bubbles, halos, light refractions, or CK-positive debris.

The cellular channel type classifications were assigned via a machine learning methodology. Three binary machine learning models were used to determine channel positivity for the Vim, CK, and CD45/CD31 channels. The input for the machine learning models included 297 cell level parameters, which constitute a subset of the 761 morphometric parameters derived using the channel in question. The slide level and frame level average and standard deviations of the 297 parameters were also added as inputs to the model, to include frame and slide level variability. Each model was trained and tested on 15,530 previously manually annotated cells from 514 slides. The random forest architecture from python library scikit-learn version 0.23.2 was used to develop the machine learning models.

### Statistical analysis models

To determine the correlations between time and location parameters and rare events detected in the liquid biopsy samples, non-parametric statistical tests were used, with *p*-values below 0.05 considered as statistically significant. The Spearman’s rank correlation^41^ was used to identify associations between rare event categories, while the Mann–Whitney U^42^, or Wilcoxon rank-sum^43^ test was applied to compare NDs with PDAC patients. For paired samples, matched by locations and draw times, the Wilcoxon signed-rank test was used^43^. The two-sided test was performed to obtain the *p*-values. For the statistically significant cases, the one-sided tests were subsequently performed solely to assess whether the difference was due to an increase or decrease in liquid biopsy analyte detection. To perform correlation analysis of liquid biopsy analytes with clinical data elements, Wilcoxon rank-sum test was used for categorical clinical variables and Spearman’s rank correlation was used for continuous and ordinal clinical variables.

Cellular morphometrics were used to investigate the heterogeneity of the rare-event population. Cell heterogeneity was visualized via morphometric probability distributions and a two-dimensional plane visualization created by a dimensionality reduction algorithm. To generate the cellular tSNE (t-distributed stochastic neighbor embedding), the median intensity of the four channels was used as well as the area, eccentricity of the cells and nucleus. Furthermore, each point was color-coded according to channel-type classification.

All computational analyses and visualizations were performed in python (version 3.8.5, https://www.python.org/) utilizing the scipy (version 1.5.0, https://scipy.org/), scikit-learn (version 0.23.2, https://scikit-learn.org/stable/), and matplotlib (version 3.2.2, https://matplotlib.org/) packages. For visualizing the variation of morphological parameters of detected cellular events and to uncover their variation within the channel-based classification, a two-dimensional tSNE was used^44^.

### Reporting summary

Further information on research design is available in the Nature Research Reporting Summary linked to this article.

### Supplementary information

REPORTING SUMMARY

Supplemental Table 1

## Data Availability

All data discussed in this manuscript are included in the main manuscript text or supplementary materials. The imaging data are available through the BloodPAC Data Commons, Accession ID “BPDC000134”.

## Code availability

All code developed is standard and easily reproducible.
