# Supplementary material for: Cancer-related cells and oncosomes in the liquid biopsy of pancreatic cancer patients undergoing surgery
Source: NPJ Precis Oncol. 2024 Feb 15;8:36. doi: 10.1038/s41698-024-00521-0 (PMC10869814; doi:10.1038/s41698-024-00521-0)
Supplement: Supplementary file 2 — Supplemental Table 1 [file 41698_2024_521_MOESM2_ESM.pdf]

**Supplemental Table 1.** HDSCA3.0 liquid biopsy rare event counts/mL for each sample provided as median, (mean), (range). D: DAPI, V: Vim, CD: CD45/CD31.

| Rare event counts per mL | ND | PB | | | PoVB | | |
|---|---|---|---|---|---|---|---|
| | | Pre | During | Post | Pre | During | Post |
| Total Rare Event | 27.55<br>(39.70)<br>(4.39-132.94) | 67.14<br>(205.98)<br>(1.1-1694.53) | 73.28<br>(141.98)<br>(14.34-447.53) | 100.80<br>(220.35)<br>(24.89-1225.53) | 74.41<br>(146.49)<br>(17.39-575.85) | 75.67<br>(221.91)<br>(11.81-1980.01) | 137.44<br>(344.87)<br>(25.05-2609.54) |
| Total Rare Cell | 11.5<br>(2.53)<br>(0-16.33) | 57.00<br>(198.45)<br>(7.17-1672.98) | 21.5<br>(34.81)<br>(5-134.5) | 92.91<br>(142.42)<br>(12.08-501.91) | 62.78<br>(130.32)<br>(11.37-562.04) | 67.73<br>(106.69)<br>(3.95-587.24) | 104.80<br>(259.17)<br>(20.51-1902.09) |
| Total CK+ Cell | 7.14<br>(15.30)<br>(0-102.90) | 27.92<br>(87.93)<br>(0-782.90) | 22.63<br>(57.36)<br>(0-365.21) | 24.51<br>(77.70)<br>(3.38-490.64) | 41.85<br>(87.80)<br>(0 - 534.45) | 31.72<br>(61.17)<br>(1.16-495.02) | 63.28<br>(176.67)<br>(0-1749.50) |
| Epi.CTC (DAPI\|CK) | 0<br>(0.24)<br>(0-4.87) | 0<br>(2.24)<br>(0-38.99) | 0<br>(0.86)<br>(0-12.41) | 0<br>(0.56)<br>(0-6.47) | 0<br>(10.9)<br>(0-158.47) | 0<br>(0.80)<br>(0-4.07) | 0<br>(51.68)<br>(0-762.26) |
| Mes.CTC (DAPI\|Vim\|CK) | 0<br>0.82<br>(0-17.12) | 0<br>(0.83)<br>(0- 6.50) | 0<br>(0.66)<br>(0 -2.57) | 0<br>(1.134)<br>(0- 11.01) | 0<br>(2.55)<br>(0-26.95) | 0<br>(0.83)<br>(0-3.76) | 0<br>(51.08)<br>(0-801.90) |
| DAPI\|CK\|Vim\|CD45/CD31 | 4.84<br>(9.57)<br>(0-57.87) | 26.03<br>(25.33)<br>(4.96-72.0) | 18.95<br>(54.19)<br>(0-357.52) | 21.71<br>(69.09)<br>(0-406.33) | 37.56<br>(67.59)<br>(0-457.31) | 29.19<br>(56.48)<br>(1.16-470.94) | 35.38<br>(60.99)<br>(0-261.46) |
| DAPI\|CK\|CD45/CD31 | 0<br>4.61<br>(0-81.56) | 0.84<br>(6.31)<br>(0- 37.65) | 0<br>(1.64)<br>(0-10.20) | 0<br>(6.91)<br>(0-84.31) | 2.48<br>(6.70)<br>(0-55.32) | 0.54<br>(3.05)<br>(0-21.68) | 1.88<br>(12.92)<br>(0-156.40) |
| DAPI\|Vim\|CD45/CD31 | 3.55<br>8.89<br>(0-70.61) | 1.53<br>(80.78)<br>(0-1488.0 | 2.07<br>(8.39)<br>(0-87.78) | 2.30<br>(3.57)<br>(0-12.60) | 1.67<br>(2.72)<br>(0-11.74) | 2.62<br>(5.07)<br>(0-20.06) | 5.26<br>(8.53)<br>(0-53.19) |
| DAPI\|Vim | 3.01 | 19.62 | 31.28 | 25.14 | 17.31 | 17.70 | 19.88 |

| | | | | | | | |
|---|---|---|---|---|---|---|---|
| | (6.28)<br>(0-75.11) | (25.33)<br>(4.96-71.97) | (46.01)<br>(4.06-234.60) | (55.62)<br>(0-291.80) | (36.46)<br>(7.86-312.32) | (35.19)<br>(0-205.91) | (67.52)<br>(0-545.31) |
| DAPI\|CD45/CD31 | 0<br>2.29<br>(0-28.63) | 0<br>(0.85)<br>(0- 4.43) | 0<br>(1.64)<br>(0-10.20) | 0<br>(1.23)<br>(0-11.28) | 0<br>(0.77)<br>(0-4.58 | 0<br>(0.85)<br>(0-5.58) | 0<br>(1.30)<br>(0-15.57) |
| DAPI-only | 3.03<br>(4.49)<br>(0-27.08) | 0<br>(3.57)<br>(0-26.75). | 2.01<br>(3.80)<br>(0-18.66) | 2.81<br>(4.29)<br>(0-23.50) | 2.69<br>(2.57)<br>(0-9.73) | 1.95<br>(4.42)<br>(0-40.89) | 2.88<br>(5.15)<br>(0-29.72) |
| Total Oncosomes | 0<br>0.28<br>(0-6) | 3.90<br>(7.53)<br>(0-23.90) | 5.03<br>(25.30)<br>(0-232.98) | 8.64<br>(77.93)<br>(0-1080.33) | 7.915<br>(16.18)<br>(0-122.81) | 3.155<br>(115.22)<br>(0-1828.28) | 16.59<br>(85.709)<br>(0-707.45) |
| CK onc. | 0<br>0.49<br>(0-14.56) | 0.85<br>(2.44)<br>(0-11.79) | 1.51<br>(2.63)<br>(0-10.32) | 0.88<br>(3.71)<br>(0-14.17) | 1.27<br>(2.44)<br>(0-7.83) | 1.82<br>(3.11)<br>(0-16.23) | 1.28<br>77.42)<br>0-689.16) |
| Vim\|CK onc. | 0<br>0.02<br>(0-2.36) | 0<br>(0.09)<br>(0-0.88) | 0<br>(0.38)<br>(0-3.47) | 0<br>(0.96)<br>(0-10.94) | 0<br>(0.44)<br>(0-3.90) | 0<br>(1.00)<br>(0-12.07) | 0<br>(1.29)<br>0-12.35) |
| CK\|CD45/CD31 onc. | 0<br>0.18<br>(0-10.59) | 0<br>(0.56)<br>(0-2.45) | 0<br>(1.51)<br>(0-10.26) | 0<br>(1.23)<br>(0-10.86) | 0<br>(0.66)<br>(0-5.91) | 0<br>(1.17)<br>(0-12.34) | 0<br>2.79)<br>0-25.83) |
| CK\|Vim\|CD45/CD31 onc. | 0<br>0.08<br>(0-2.75) | 0<br>(4.44)<br>(0-21.55) | 1.70<br>(20.77)<br>(0-224.72) | 3.20<br>(72.04)<br>(0-1071.81) | 2.00<br>(12.640)<br>(0-120.30) | 1.05<br>(109.93)<br>(0-1808.22) | 1.79<br>(4.19)<br>(0-18.29) |

**Supplemental Table 2.** P-values for the comparison of liquid biopsy analytes between different cohorts (ND vs. PDAC Pre-Surgery PB), timepoints (Pre-, During-, Post-surgery), and anatomical locations (PB vs. PoVB). D: DAPI, V: Vim, CD: CD45/CD31.

| **Rare Event** | **ND vs PDAC** | **PB** | | | **PoVB** | | | **PB vs PoVB** | | |
|---|---|---|---|---|---|---|---|---|---|---|
| | | **Pre vs During** | **Pre vs Post** | **During vs Post** | **Pre vs During** | **Pre vs Post** | **During vs Post** | **Pre** | **During** | **Post** |
| Total Rare Event | **0.001** | 0.832 | 0.312 | 0.081 | 0.953 | 0.104 | **0.002** | 0.490 | 0.865 | 0.154 |
| Total Rare Cell | **0.001** | 0.865 | 0.418 | 0.325 | 0.709 | 0.595 | **0.008** | 0.922 | 0.442 | 0.580 |
| Total CK+ Cell | **0.025** | 0.687 | 0.922 | 0.369 | 0.182 | 0.879 | **0.027** | 0.586 | 0.832 | 1.000 |
| Epi.CTC (DAPI\|CK) | 0.747 | 0.753 | 0.893 | 1.000 | 0.182 | 0.722 | 0.139 | 0.248 | 0.327 | **0.025** |
| Mes.CTC (DAPI\|Vim\|CK) | 0.564 | 0.889 | 0.953 | 0.678 | 0.814 | 0.929 | 0.110 | 0.424 | 0.859 | 0.374 |
| DAPI\|CK\|Vim\|CD45/CD31 | **0.009** | 0.653 | 0.879 | 0.309 | 0.490 | 0.446 | 0.167 | 0.845 | 0.932 | 0.347 |
| DAPI\|CK\|CD45/CD31 | 0.768 | 0.196 | 0.776 | **0.050** | 0.191 | 0.918 | 0.463 | 0.753 | 0.859 | 0.279 |
| DAPI\|V\|CD45/CD31 | 0.101 | 0.877 | 0.836 | 0.730 | 0.125 | **0.044** | 0.345 | 0.906 | 0.776 | 0.064 |
| DAPI\|Vim | **0.000** | 0.154 | **0.023** | 0.671 | 1.000 | 0.798 | 0.054 | 1.000 | 0.119 | 0.586 |
| DAPI\|CD45/CD31 | 0.053 | 0.721 | 0.790 | 0.859 | 0.314 | 0.767 | 0.767 | 0.328 | 0.767 | 0.859 |
| DAPI-only | **0.007** | 0.221 | 0.281 | 0.831 | 0.554 | **0.044** | 0.332 | 0.570 | 0.756 | **0.039** |
| Total Oncosomes | **0.000** | 0.108 | **0.022** | 0.181 | 0.619 | 0.215 | 0.098 | 0.170 | 0.899 | 0.795 |
| CK onc. | 0.058 | 0.836 | 0.281 | 0.730 | 0.820 | 0.501 | 0.422 | 0.691 | 0.955 | 0.861 |
| Vim\|CK onc. | 0.596 | 0.144 | 0.080 | 0.225 | 0.249 | 0.263 | 0.484 | 0.068 | 0.463 | 0.612 |
| CK\|CD45/CD31 onc. | 0.053 | 0.123 | 0.594 | 0.327 | 0.674 | 0.594 | 0.241 | 0.953 | 0.575 | 0.333 |
| CK\|Vim\|CD45/CD31 onc. | 0.005 | 0.300 | **0.020** | 0.281 | 0.311 | 0.638 | 0.248 | 0.221 | 0.861 | 0.917 |

**Supplemental Table 3.** Correlation of HDSCA3.0 liquid biopsy analytes with pathological T stage and tumor volume. Significant differences are indicated in bold text. D: DAPI, V: Vim, CD: CD45/CD31.

| Rare Event | pT stage | | Tumor Volume | |
|---|---|---|---|---|
| | PB | PoVB | PB | PoVB |
| Total Rare Event | 0.618 | 0.110 | **0.036** | **0.047** |
| Total Rare Cell | 0.731 | 0.104 | **0.036** | **0.010** |
| Total CK+ Cell | **0.015** | **0.019** | **0.014** | 0.058 |
| Epi.CTC (DAPI\|CK) | 0.130 | 0.383 | | 0.604 |
| Mes.CTC (DAPI\|Vim\|CK) | 0.914 | 0.877 | 0.661 | 0.558 |
| DAPI\|CK\|Vim\|CD45/CD31 | **0.038** | **0.031** | 0.052 | **0.021** |
| DAPI\|CK\|CD45/CD31 | **0.010** | **0.045** | 0.159 | 0.509 |
| DAPI\|Vim\|CD45/CD31 | 0.776 | 0.660 | 0.760 | 0.863 |
| DAPI\|Vim | 0.066 | 0.725 | 0.482 | **0.015** |
| DAPI\|CD45/CD31 | 0.612 | 0.703 | 0.491 | 0.452 |
| DAPI-only | 0.514 | 0.932 | 0.240 | 0.601 |
| Total Oncosomes | 0.707 | 0.750 | 0.670 | 0.365 |
| CK onc. | 0.756 | 0.480 | 0.867 | 0.608 |
| Vim\|CK onc. | 0.894 | 0.237 | | 0.604 |
| CK\|CD45/CD31 onc. | 0.900 | 0.215 | 0.363 | 0.314 |
| CK\|Vim\|CD45/CD31 onc. | 0.632 | 0.960 | 0.702 | 0.365 |
